# Uptake and function of membrane‐destabilizing cationic nanogels for intracellular drug delivery

**DOI:** 10.1002/btm2.10120

**Published:** 2018-11-22

**Authors:** William B. Liechty, Rebekah L. Scheuerle, Julia E. Vela Ramirez, Nicholas A. Peppas

**Affiliations:** ^1^ McKetta Dept. of Chemical Engineering The University of Texas at Austin Austin TX 78712; ^2^ Dept. of Biomedical Engineering The University of Texas at Austin Austin TX 78712; ^3^ Institute for Biomaterials, Drug Delivery, and Regenerative Medicine The University of Texas at Austin Austin TX 78712; ^4^ Depts. of Surgery and Perioperative Care Dell Medical School, The University of Texas at Austin Austin TX 78712; ^5^ Division of Molecular Pharmaceutics and Drug Delivery College of Pharmacy, The University of Texas at Austin Austin TX 78712

**Keywords:** cationic, drug delivery, intracellular, nanoparticles, polymer

## Abstract

The design of intracellular drug delivery vehicles demands an in‐depth understanding of their internalization and function upon entering the cell to tailor the physicochemical characteristics of these platforms and achieve efficacious treatments. Polymeric cationic systems have been broadly accepted to be membrane disruptive thus being beneficial for drug delivery inside the cell. However, if excessive destabilization takes place, it can lead to adverse effects. One of the strategies used to modulate the cationic charge is the incorporation of hydrophobic moieties, thus increasing the hydrophobic content. We have demonstrated the successful synthesis of nanogels based on diethylaminoethyl methacrylate and poly(ethylene glycol) methyl ether methacrylate. Addition of the hydrophobic monomers *tert*‐butyl methacrylate or 2‐(*tert*‐butylamino)ethyl methacrylate shows improved polymer hydrophobicity and modulation of the critical swelling pH. Here, we evaluate the cytocompatibility, uptake, and function of these membrane‐destabilizing cationic methacrylated nanogels using in vitro models. The obtained results suggest that the incorporation of hydrophobic monomers decreases the cytotoxicity of the nanogels to epithelial colorectal adenocarcinoma cells. Furthermore, analysis of the internalization pathways of these vehicles using inhibitors and imaging flow cytometry showed a significant decrease in uptake when macropinocytosis/phagocytosis inhibitors were present. The membrane‐disruptive abilities of the cationic polymeric nanogels were confirmed using three different models. They demonstrated to cause hemolysis in sheep erythrocytes, lactate dehydrogenase leakage from a model cell line, and disrupt giant unilamellar vesicles. These findings provide new insights of the potential of polymeric nanoformulations for intracellular delivery.

## 1 INTRODUCTION

Particle transport across the cellular membrane is a complex process due to the presence of lipid bilayers that prevent the permeation of exogenous molecules.^1^, ^2^ These sheet‐like structures are formed by thousands of lipid molecules maintained together by hydrophobic interactions.^1^ In particular, for mammalian cell membranes, there are three main types of lipids: glycerophospholipids, sphingolipids, and cholesterol.^1^, ^2^ Variation on the arrangement of these molecules confers different physicochemical properties to the membrane (i.e., fluidity, electric charge, molecular weight), which makes entering the cytosol highly difficult.^1^, ^2^, ^3^

Cellular internalization of polymeric nanoparticles for drug delivery applications is a complex phenomenon that depends on size,^4^, ^5^ shape,^6^, ^7^ polymer chemistry,^8^ and surface characteristics of drug delivery carriers.^9^ Specifically, for intracellular delivery, understanding the mechanism of uptake is critical because the internalization pathway influences subcellular trafficking, sorting, and exposure to variable enzymatic and pH conditions.^10^, ^11^, ^12^ Additionally, compositional considerations of the particle chemistry, such as the balance between cationic and nonionic, hydrophilic components, and ratio of hydrophobic monomers have significant impact on resultant drug delivery properties (i.e., transfection efficiency, complex stability, etc.).^13^ These parameters must be carefully investigated and optimized in the development of polymer drug delivery systems.

It is generally understood that increasing cationic content leads to increased loading efficiency of negatively charged molecules.^14^, ^15^, ^16^ Previous work on the interaction between poly(dimethylaminoethyl methacrylate; PDMAEMA) or poly(aminoethyl methacrylate; PAEMA) and negatively‐charged molecules (i.e.*,* DNA) showed that PAEMA interacts more strongly with DNA while PDMAEMA exhibited superior buffering capacity,^13^ which could lead to increased endosomolytic activity. However, excess cationic content in polymeric delivery systems can have deleterious effects. High cationic charge density is frequently correlated with toxicity of conventional cationic polymers like poly(ethyleneimine)^17^ and may host undesirable consequences in vivo.^18^

The synthetic strategy developed by Peppas and coworkers^19^, ^20^, ^21^ allowed the decrease of critical swelling pH to increase endosomolytic^22^ and gene transfection^23^ efficiency by the incorporation of *tert*‐butyl methacrylate (TBMA) in a cationic nanogel formulation. Previous studies demonstrated the pH‐dependent aqueous solution behavior of P(DEAEMA‐*g*‐PEGMA; PDET) and P(DEAEMA‐co‐TBMA‐*g*‐PEGMA; PDETB) nanogels.^21^ TBMA‐modified networks exhibited a more tightly collapsed network at elevated pH,^19^ which could theoretically provide improved protection of encapsulated payload. Moreover, a decrease in the pH required to induce a critical transition (as demonstrated by PDETB30) may minimize premature release of the cargo before the intended site of action.^21^

The work presented herein analyzes the role of hydrophobicity in modulating the cytotoxicity, uptake, and membrane destabilization properties of polymeric cationic nanogels. The hydrophobic monomers TBMA and 2‐(*tert*‐butylamino)ethyl methacrylate (TBAEMA) were incorporated to the base formulation of PDET. The toxicity of the synthesized drug delivery vehicles was evaluated using a mammalian cell model. Mechanistic studies using imaging flow cytometry were then performed to identify the particle internalization pathways to elucidate the principal cellular mechanisms used for their uptake.^24^ Finally, evaluation of the membrane disruption abilities of these drug delivery vehicles was performed using three model membrane systems.

Sheep erythrocytes were used to assess the pH‐ and concentration‐dependent membrane destabilization of lipid bilayers, lactate dehydrogenase (LDH) leakage was measured from Caco‐2 cells to evaluate the nonspecific membrane destabilization in live cells, and giant unilamellar vesicles (GUVs) were used to understand the mechanism of membrane disruption by cationic polymeric nanogels.^25^ The insights obtained from these studies will help in the design of novel drug delivery platforms for intracellular delivery by understanding the role of charge and hydrophobicity in the uptake and function of cationic nanoparticle platforms.

## 2 MATERIALS AND METHODS

### 2.1 Polymer synthesis and purification

Polymer synthesis and purification proceeded as described previously.^21^, ^26^ Briefly, 2‐(diethylamino) ethyl methacrylate (DEAEMA, Sigma‐Aldrich, St. Louis, MO), TBAEMA (Polysciences, Inc., Warrington, PA), *tert*‐butyl methacrylate (TBMA, Sigma‐Aldrich, St. Louis, MO), and tetra(ethylene glycol) dimethacrylate (TEGDMA, Sigma‐Aldrich, St. Louis, MO) were passed through a column of basic alumina powder to remove inhibitor prior to use. Poly(ethylene glycol) methyl ether methacrylate (PEGMA), Mn ~ 2080, (Sigma‐Aldrich, St. Louis, MO) was used as received. DEAMA, TEGDMA, and TBMA or TBAEMA were added to an aqueous solution containing 5 wt% PEGMA, Irgacure 2959 (Ciba Geigy, Tarrytown, NY) at 0.5 wt% of total monomer, 4 mg ml^−1^ Brij‐30 (Acros Ogranics, Fair Lawn, NJ) and ionic surfactant myristyltrimethylammonium bromide (MyTAB, Sigma‐Aldrich, St. Louis, MO). The reaction pH was kept at pH 8.5. The mixture was emulsified using a Misonix Ultrasonicator (Misonix, Inc., Newtown, CT). The emulsion was purged with nitrogen gas and exposed to a UV source for 2.5 hr with constant stirring. MyTAB, Brij 30, and unreacted monomers were then removed by repeatedly inducing polymer‐ionomer collapse, separating particles by centrifugation, and resuspending in 0.5 N HCl. Polymer particles were dialyzed against ddH20 for 7 days with twice daily water changes. Following dialysis, polymeric particles were flash frozen in liquid N2 and lyophilized for 5 days. Transmission electron microscopy was used to determine the diameter of the dry nanogels and was conducted as previously described.^21^

### 2.2 Cell culture

Human colorectal adenocarcinoma cells (Caco‐2) were maintained in Dulbecco's Modified Eagle's Medium (DMEM) supplemented with 100 U ml^−1^ penicillin, 100 μg ml^−1^ streptomycin, 0.25 μg ml^−1^Amphotercin B, and 10% FBS. Caco‐2 cells were used between passage 34 and 62. Cells were passaged by washing with prewarmed Dulbecco's phosphate buffered saline (DPBS) and subsequent incubation with 0.25% Trypsin–EDTA at 37 °C. Trypsin was neutralized by addition of fresh DMEM and cells were separated by centrifugation (10 min, 100 rcf). Caco‐2 cells were passaged at 1:5 ratio with media replenished every 2–3 days.

### 2.3 Cytocompatibility studies

In vitro cytocompatibility of polycationic nanoscale hydrogels was evaluated using commercially available cytotoxicity assays. MTS assays were performed using the CellTiter 96 AQueous One Solution Cell Proliferation Assay kit (Promega Corp., Madison, WI) in which the soluble tetrazolium salt (3‐[4,5‐dimethylthiazol‐2‐yl]‐5‐[3‐carboxymethoxyphenyl]‐2‐[4‐sulfophenyl]‐2H‐tetrazolium; MTS) is reduced to a purple formazan product. The absorbance of the formazan product is proportional to the number of viable cells. Stock solutions of polymer were suspended in PBS and allowed to equilibrate overnight. Caco‐2 cells were seeded in 96‐well plates at 15,000 cells/well in 200 μl DMEM for 36 hr prior to the assay. Media was aspirated and cells were washed twice with DPBS and incubated in 160 μl of serum‐free DMEM for 90 minutes. Following this incubation period, polymer stock solutions at 5X were added to cells for designated exposure times. Media and polymer were aspirated and replaced with a DMEM/MTS solution. Absorbance at 490 nm was recorded after 4 hr incubation.

### 2.4 Fluorescent polymer synthesis

PDETB30 was synthesized and purified as described above. To enable the covalent conjugation of a fluorescent probe, 2‐aminoethyl methacrylate hydrochloride (AEMA) was included in the pre‐polymer feed mixture at 5 mol% of DEAEMA. The resulting copolymer was named PDETB30f to signify the amine functionality. The primary amine of AEMA was verified with a fluorescamine assay after synthesis and purification.^19^

Oregon Green 488 carboxylic acid, succinimidyl ester (OG488) was purchased from Molecular Probes (Eugene, OR). The solid dye was dissolved in DMSO to yield a 10 mg ml^−1^ solution. To form the fluorescent polymer conjugate, PDETB30f was suspended at 10 mg ml^−1^ in 150 mM sodium bicarbonate buffer, pH 8.30. OG488 was added to the PDETB30f suspension to give a 1:1 mol ratio between AEMA and OG488. The reaction was stirred in the dark for 6 hr. Following reaction completion, unreacted dye was separated from labeled PDETB30f through dialysis against DI water. Dialysis proceeded for 3 days using 12,000–14,000 MWCO dialysis tubing (Spectrum Labs, Rancho Dominguez, CA) for 3 days. Labeled nanogels, PDETB30‐OG488, were lyophilized in the dark for 3 days.

### 2.5 Mechanism of nanogel uptake

Chlorpromazine hydrochloride (98%), Nystatin, and Wortmannin (98%) were purchased from Sigma‐Aldrich (St. Louis, MO). 5‐N,N‐dimethyl amiloride was purchased from Enzo Life Sciences (Farmingdale, NY). Filipin III was purchased from Cayman Chemical (Ann Arbor, MI). Inhibitor toxicity to Caco‐2 cells was evaluated using MTS assays as previously described.

Caco‐2 cells were seeded at 1 x 10^5^ cells/well in 6‐well plates and allowed to grow to 80% confluence before performing the experiment. Immediately prior to exposure to inhibitors, cells were washed with 2 ml DPBS and media was replaced with 1.8 ml serum‐free DMEM. Concentrated suspensions (20X) of inhibitors were added to wells in 100 μl increments and allowed to incubate with cells for 30 min in a 37 °C, 5% CO_2_ atmosphere. Cells inhibited by refrigeration were placed at 4 °C for 30 min prior to nanogel exposure.

Following the 30 min equilibration period, 100 μl of PDETB30‐OG488 at 500 μg ml^−1^ in PBS was added to test well to yield a final concentration of 25 μg ml^−1^. Control wells received 100 μl PBS. Nanogel exposure occurred for 60 min at 37 °C or 4 °C. Following the exposure period, cells were rinsed 3× DPBS (with calcium and magnesium) and the media was replaced with 2 ml serum‐free DMEM. Hoechst 33342 was added to each well for nuclear staining at a final concentration of 2.5 μg ml^−1^. The nuclear staining process was completed for 45 min at 37 °C, 5% CO_2_. Following Hoechst incubation, cells were rinsed 3× with DPBS (without calcium and magnesium).

Caco‐2 cells were isolated by replacing the final DPBS wash with 500 μl 0.25% trypsin–EDTA and incubating at 37 °C, 5% CO_2_ for 8 min. Trypsin was neutralized by adding 3 ml DMEM with 10% FBS and without phenol red. Cell suspensions were centrifuged for 5 min at 500g. The supernatant was discarded and cell pellet resuspended in 100 μl flow cytometry buffer. All cell suspensions were kept on ice until analysis with Image Stream Cytometry. Propidium iodide (PI) was used as a live/dead discriminator and was added to cell suspensions immediately before analysis at a final concentration of 1 μg ml^−1^.

### 2.6 Imaging flow cytometry

Analysis of uptake mechanisms was conducted using an Amnis ImageStream (Seattle, WA) imaging flow cytometer equipped with lasers at 405, 488, 658, and 785 nm. For uptake studies, fluorescent data were collected using Channel 1 (430–505 nm, Hoechst), Channel 2 (505–595 nm, OG488), Channel 4 (595–660 nm, PI), and Channel 6 (745–800 nm, side scatter). Brightfield images were collected in Channel 5. Cells were imaged with a 60× objective. Fluid velocity was set to a nominal value of 40 mm/s. Fluorescent compensation matrices were constructed using Amnis IDEAS software and verified manually for proper fit. At least 5,000 cells were collected for analysis. Dead cells (PI positive) were excluded from analysis. Out‐of‐focus cells were also excluded from further analysis by gating the Gradient RMS feature in IDEAS software. This feature detects image sharpness by calculating large changes in pixel values across the brightfield image. Typically, cells with Gradient RMS value <40 were considered out of focus.

### 2.7 Hemolysis

Sheep blood in sodium citrate was obtained from Hemostat Laboratories (Dixon, CA) and used for up to 2 weeks after receipt. Phosphate buffers (150 mM) from pH 5.0 to 8.0 were prepared. Dry nanogels were suspended in 150 mM phosphate buffer at the desired pH at a concentration of 2.5 mg ml^−1^ and allowed to equilibrate overnight. Erythrocytes were isolated from whole sheep blood by three successive washes with freshly prepared 150 mM NaCl. Red blood cells (RBCs) were separated by centrifugation from 10 min at 2,000g. The supernatant was carefully aspirated and discarded. After removing the supernatant following the final wash, RBCs were suspended in a volume of 150 mM phosphate buffer identical to that of the original blood aliquot at the pH matching that of the suspended polymers. This solution was diluted 10‐fold in 150 mM phosphate buffer to yield an RBC suspension of approximately 5 x 10^8^ cells/ml. In a typical experiment, 1 x 10^8^ RBCs were exposed to nanogels at specified concentrations while shaking in a bead bath (LabArmor, Cornelius, OR) pre‐equilibrated at 37 °C. Following a 60 min incubation period, samples were centrifuged at 14,500 RPM for 5 min to separate cells and membrane fragments. An aliquot of each sample was transferred to a clear 96‐well plate and hemoglobin absorbance was measured at 541 nm. Negative controls (0% lysis) consisted of 150 mM phosphate buffer at experimental pH and positive controls (100% lysis) consisted of RBCs incubated in ultrapure DI water.

The pH values tested in this analysis range from pH 5.0 to 8.0; experiments performed at pH 5.00, 5.50, 6.00, 6.50, 7.40, 7.60, 7.80, and 8.00. The concentrations tested range from 1 to 2,000 μg ml^−1^; with experiments performed with 2,000, 1,000, 500, 250, 100, 50, 25, 10, 5, 2.5, and 1 μg ml^−1^ nanogel suspended in 150 mM phosphate buffer at the specified pH.

### 2.8 Pyrene fluorescence

Pyrene (Puriss grade, >99.0%, Sigma‐Aldrich, St. Louis, MO) was used as received from the manufacturer. Phosphate buffer solutions from pH 5.8 to pH 8.0 were prepared by combining solutions of 200 mM NaH_2_PO_4_ • H_2_O and 200 mM Na_2_HPO_4_ • 7H_2_O. Polymer solutions were prepared in DI water at a concentration of 1 mg ml^−1^. These two solutions were mixed in equal volumes to give a final concentration of nanoparticles of 0.5 mg ml^−1^ in 100 mM phosphate buffer. Pyrene was dissolved in methanol at 1 mM. Fluorescence spectra were collected on a Fluorlog‐3 Spectrofluorometer (Jobin Yvon, Horiba Scientific, Edison, NJ). Emission spectra were collected with λ_ex_ = 339 nm, 1 nm increments, 1.5 nm slit with for excitation, 1 nm slit width for emission, and 0.8 s integration time. Excitation spectra were collected with λ_em_ = 390 nm, 1 nm increments, 1 nm slit for excitation, 1.5 nm for emission, and 0.8 s integration time.

### 2.9 Lactate dehydrogenase release

LDH assays were performed using a CytoTox‐ONE Homogeneous Membrane Integrity Assay (Promega Corp., Madison, WI) to measure release of lactate dehydrogenase (LDH) from cells with damaged membranes. Cells were seeded to 96‐well plates and polymer solutions added as previously described. At designated time points, 50 μl aliquots of media was aspirated and combined with 50 μl LDH assay buffer in a black‐walled 96‐well plate. Following 10 minutes incubation at room temperature, the fluorescence was measured at 530 ex/590 em. Generally, cell culture plates were used for a maximum of two different aliquots.

### 2.10 Giant unilamellar vesicle disruption

1‐palmitoyl‐2‐oleoyl‐sn‐glycero‐3‐phosphocholine (POPC), 1,2‐dihexadecanoyl‐sn‐glycero‐3‐phosphoethanolamine (DHPE) labeled with BODIPY FL, cholesterol, and Texas Red‐sucrose were kindly donated by Prof. Jeanne Stachowiak (University of Texas at Austin, Austin, TX). Giant Unilamellar Vesicles (GUVs) were synthesized via electroformation as previously described.^27^, ^28^ Briefly, lipid/cholesterol solutions were combined in the following ratio: 7:3:0.01 POPC : Cholesterol : Bodipy FL DHPE and drop‐cast onto clean glass slides. The lipid solutions were allowed to dry and were then assembled into electroformation chambers. Vesicles were electroformed at 60 °C in Texas Red‐sucrose (∼350 milliosmole[mOsm]) solution.

GUVs were placed in 35 mm glass‐bottom petri dishes for real‐time confocal microscopy imaging. PDET and PDETB30 were prepared at 2 mg ml^−1^ in 100 mM phosphate buffer adjusted to pH 6.50. The osmolarity of the resulting suspensions was measured and adjusted with sucrose to ~350 mOsm as needed. 1 ml of GUV suspension was transferred to the glass‐bottom petri dish and was allowed to sediment for 5 min. About 25 μl of the nanogel suspension was carefully injected into the dish so as not to disturb the spatial distribution of focused GUVs. Images were collected every 5 s at a fixed focal plane.

### 2.11 Statistical analysis

Statistical comparisons between experimental and control groups were made with two‐tailed, unpaired, Student's *t*‐test. Differences were accepted as statistically significant with *p* < .05.

## 3 RESULTS AND DISCUSSION

Our previous work has demonstrated the ability to synthesize cationic polymeric nanogels based on a core of PDEAEMA using a photoemulsion polymerization.^19^, ^20^, ^21^ Furthermore, we were able to tailor their hydrophobicity by the incorporation of the hydrophobic monomers TBMA and TBAEMA.^21^, ^26^ The physicochemical characteristics of the resulting particles displayed promising potential as drug delivery vehicles including their size, pH‐responsiveness, and swelling volume. Based on these results, the most promising formulations were selected to evaluate their capabilities in vitro as membrane‐destabilizing platforms for intracellular drug delivery. This work was focused on the assessment of nanogels prepared from copolymers with 0–30% mol of hydrophobic monomer (TBMA or TBAEMA).

Polycationic nanoscale hydrogels were successfully synthesized using a photoemulsion polymerization as previously described.^21^ Copolymers with TBMA or TBAEMA (0, 10, 20, 30 mol%) were formulated to increase the core hydrophobicity. The polymer formulation nomenclature includes the numerical suffix on the polymer name (e.g., PDETB30 or PDETBA20) which refers to the moles of hydrophobic monomer (TBMA or TBAEMA) per 100 mol of DEAEMA. The composition and morphology of the resulting polymeric particles were consistent with previously reported results.^21^ Representative micrographs of the synthesized nanogels are presented in Supporting Information Figure S1.

Following the synthesis of cationic nanogels, the initial step in the evaluation of their biological properties was the assessment of their cytocompatibility. These materials will interact with a variety of cellular populations upon entering the body. Hence, a critical characteristic as drug delivery vehicles is their lack of cytotoxic effects.

The influence of polymer concentration and composition on cellular proliferation was assessed using MTS assays. These data are important to determine the nontoxic polymer doses for future drug delivery experiments. In this assay, the metabolic activity of an experimental population relative to control populations can be given by the ratio:

$$\text{Relative Proliferation}=\frac{A_{s}-A_{\mathrm{bkg}}}{A_{\mathrm{PBS}}-A_{\mathrm{bkg}}}$$

where *A*_s_ is the absorbance (λ = 490 nm) from sample wells, *A*_bkg_ is the background absorbance from DMEM/MTS solution, and *A*_PBS_ is the absorbance from wells in which cells were incubated only with DPBS.

**Figure 1:**
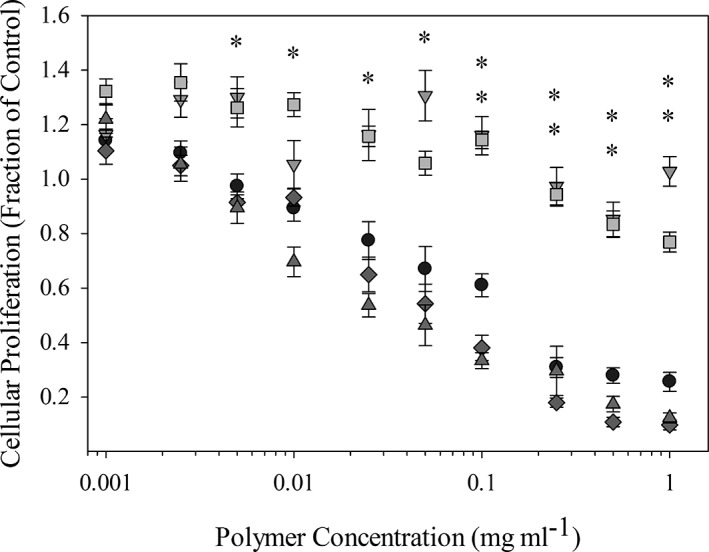
Cytocompatibility of polycationic nanogels as a function of polymer concentration. Symbols represent PDET (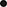), PDETB20 (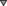), PDETB30 (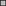), PDETBA20 (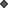), or PDETBA30 (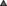). Proliferation of Caco‐2 cells was determined by MTS assay following 90 min nanogel exposure and is expressed as a fraction of the control (untreated) cells. Data are expressed as mean ± *SEM*, *n* = 8. Statistical significance determined via pairwise *t*‐test between cells exposed to PDETB20 and PDET or PDETB30 and PDET (*p < .005)

As seen in Figure 1, PDETB20 and PDETB30 were nontoxic to Caco‐2 cells at concentrations below 0.5 mg ml^−1^. Additionally, the synthesized formulations with the incorporation of TBMA or TBAEMA were significantly less toxic than the base formulation of PDET in a concentration range of 0.05–2 mg ml^−1^. It has been well documented that free amino groups contribute to the untoward cytotoxicity of many polycationic delivery agents and that increased cationic charge density correlates with increased cytotoxicity.^17^ As expected, polymers with similar cationic charge densities, for example, nanogels with 20 and 30 mol% TBAEMA, as well as PDET, exhibited similar toxicity profiles. By nature of the polymer composition, nanogels with 20 and 30 mol% TBMA have less cationic charge density and thus result in decreased toxicity. An increase in particle hydrophobicity with the incorporation of the TBMA and TBAEMA monomers can also enhance internalization of the drug carrier.^16^, ^29^ Previous studies have shown that surface hydrophobicity increases particle uptake in antigen presenting cells.^9^, ^30^, ^31^, ^32^ This phenomenon has shown to be related to opsonization, as hydrophobicity has shown to increase the amount and variety of serum proteins adsorbed onto different particle platforms.^31^, ^33^ These advantageous characteristics are important in the design of intracellular delivery vehicles, where particle internalization is critical.

For intracellular delivery systems, it is important to analyze not only uptake efficiency, but the location of the vehicles upon entering the cell. To enable visualization of nanogel subcellular localization experiments, a fluorescent version of the PDETB30 nanogel was necessary. A primary amine‐containing analogue of PDETB30, termed PDETB30f, was successfully synthesized through the inclusion of AEMA in the nanogel core. Following previous work,^34^ solid AEMA was added to the pre‐polymer mixture immediately before sonication. While this monomer is stable in its hydrochloride salt form, it readily undergoes a cyclic rearrangement to 2‐hydroxyethyl methacrylamide upon neutralization.^35^ Prior to the conjugation reaction, the primary amine content of PDETB30f was determined to be 47.5 ± 0.6 μmol g^−1^, which represents a 32% incorporation efficiency.

Oregon Green 488 (OG488), an amine reactive dye, was then successfully conjugated to the primary amines in the nanogel core. OG488 was added to PDETB30f at 1:3 mol ratio of dye to amine. Following dialysis and lyophilization, the Oregon Green 488 functionalization was tested with fluorescence spectroscopy and the functionalization percentage was calculated with UV absorbance and comparison to an Oregon Green 488 standard curve. The fluorescence emission (λ_ex_ = 465 nm) spectra of the labeled nanogel (PDETB30‐OG488) was confirmed prior to use. The fluorescent labeling was estimated at 19.1 ± 0.4 μmol g^−1^ using a standard curve of OG488 in PBS and at 17.1 μmol g^−1^ using the absorbance at 496 nm and the OG‐488 extinction coefficient (ε) of 70,000 L mol^−1^ cm^−1^.

Prior to testing the uptake inhibition of PDETB30‐OG488, the specific pharmacological inhibitors were tested for toxicity against the Caco‐2 model cell line using an MTS assay. Toxicity data for specific inhibitors is seen in Supporting Information Figure S2. To ensure inhibitory activity, the concentration used in inhibition experiments was selected as the maximum possible concentration before the onset of cytotoxicity.

Clathrin‐mediated endocytosis is a ubiquitous internalization pathway that serves as the primary mode of internalization for macromolecules.^36^ Endocytic vesicles evolving from clathrin‐coated pits will deliver their contents to early endosomes and will subsequently experience vesicular acidification. Caveolae‐mediated endocytosis occurs in membrane invaginations lined with the protein caveolae and cholesterol. Vesicles generated from caveolae‐mediated endocytosis do not undergo acidification. Macropinocytosis, similar to phagocytosis, occurs via actin‐dependent membrane protrusions. This pathway is common to many cell types and results in the formation of large macropinosomes approximately 1–5 μm in diameter.^37^ In macrophages, macropinosomes typically become acidified, shrink, and subsequently fuse with lysosomes.^38^ The fate of macropinosomes is less clear in other cell types. For example, studies of macropinosome‐endosome mixing in A431 cells revel little fluid or membrane exchange with that of conventional endosomes.^39^

Historically, the constitutive endocytic activity of the clathrin‐mediated pathway has been the most attractive mechanism for internalization. Caveolae‐mediated internalization is comparatively slow^40^ and the small vesicle size (50–60 nm) precludes the entry of many nanoparticle delivery systems. As this pathway generally avoids nonproductive lysosomal accumulation, it holds promise as target for drug delivery of biomacromolecules. Some recent evidence^41^ suggests caveolae‐mediated internalization plays a critical role in transfection efficiency of cationic polymer–DNA complexes for gene delivery.

In this study, several uptake inhibitors (Table 1) were applied to Caco‐2 cells to elucidate the primary uptake pathways into enterocytes and phagocytes, respectively. As demonstrated previously, the membrane‐disruptive activity of PDETB30 is highly dependent on environmental conditions (e.g., pH). Therefore, to exert their membrane‐disruptive effect and enable cytoplasmic delivery of encapsulate cargoes, these polybasic nanogels must be exposed to a slightly acidic environment. Some internalization pathways, such as caveolae‐mediated endocytosis and lipid raft endocytosis, do not result in vesicular acidification. Thus, these trafficking pathways are both undesirable and unproductive, as PDETB30 is far less membrane disruptive at pH values of the extracellular milieu. In contrast, pathways such as clathrin‐mediated endocytosis or macropinocytosis are preferred because of their progressive vesicular acidification; a process that will enable PDETB30 to undergo a volume phase transition and destabilize the surrounding vesicular membrane.

**Table 1:** Uptake inhibitors and their inhibitory effects

Molecule	Inhibitory effect	Concentration used	Concentrations tested
Chlorpromazine	Clathrin‐mediated endocytosis by dissociating clathrin lattice	10 μM	1–100 μM
Filipin III	Caveolae via cholesterol binding	1.5 μM	0.05–50 μM
Nystatin	Caveolae/lipid raft endocytosis	50 μg ml^−1^	1–1,000 μg ml^−1^
Wortmannin	Macropinocytosis/phagocytosis by inhibiting PI3K	100 nM	0.5–500 nM
Amiloride	Macropinocytosis via preventing Na+/H+ exchange	250 μM	1–1,000 μM

In the mechanistic studies of nanogel internalization, it was particularly important to verify that the Oregon Green 488 (OG488)‐labeled nanogels were located inside the cell rather than on the periphery. To accomplish this, two image masks were created in the cell brightfield channel. The total cell mask encompasses the entire cell contents. The cell interior mask is slightly smaller than the total cell mask and encompasses cell contents *inside* the cell membrane (Supporting Information Figure S3).

The internalization coefficient is a ratio of the OG488 fluoresence intensity *inside the cell* to the OG488 fluoresence *from the whole cell*, where the cell boundaries are determined by masks shown in Supporting Information Figure S3. The histogram is scaled so a value of 0 represents that half of the intensity is inside. Two representative images illustrate the difference between extracellular or membrane‐bound fluorescence (Internalization Coefficient <0) and intracellular fluoresence (Internalization Coefficient >0). The population formed by live, focused, single cells with internalized OG488 was used as the basis for all internalization mechanism studies.

**Figure 2:**
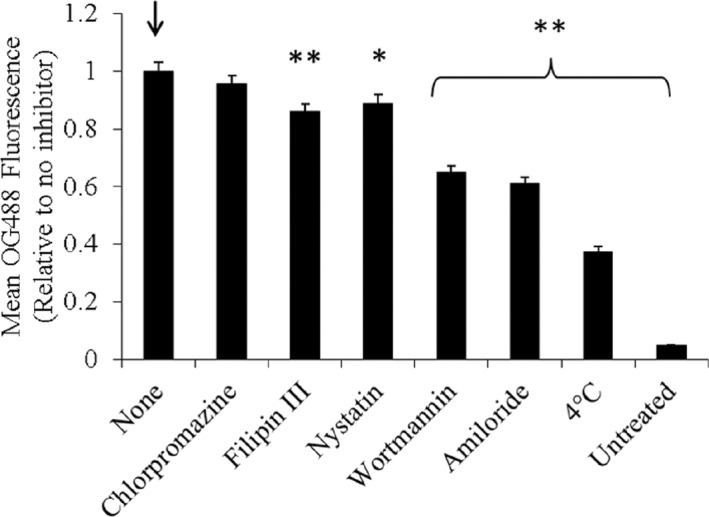
Uptake inhibition in Caco‐2 cells. Intracellular PDETB30‐OG488 fluorescence relative to noninhibited control. Caco‐2 cells preincubated with inhibitors for 30 min prior to 60 min exposure to 25 μg ml^−1^ PDETB30‐OG488. Bars represent the mean of two pooled experiments ± *SEM* *p < .05, **p < .01. Arrow designates control group

**Figure 3:**
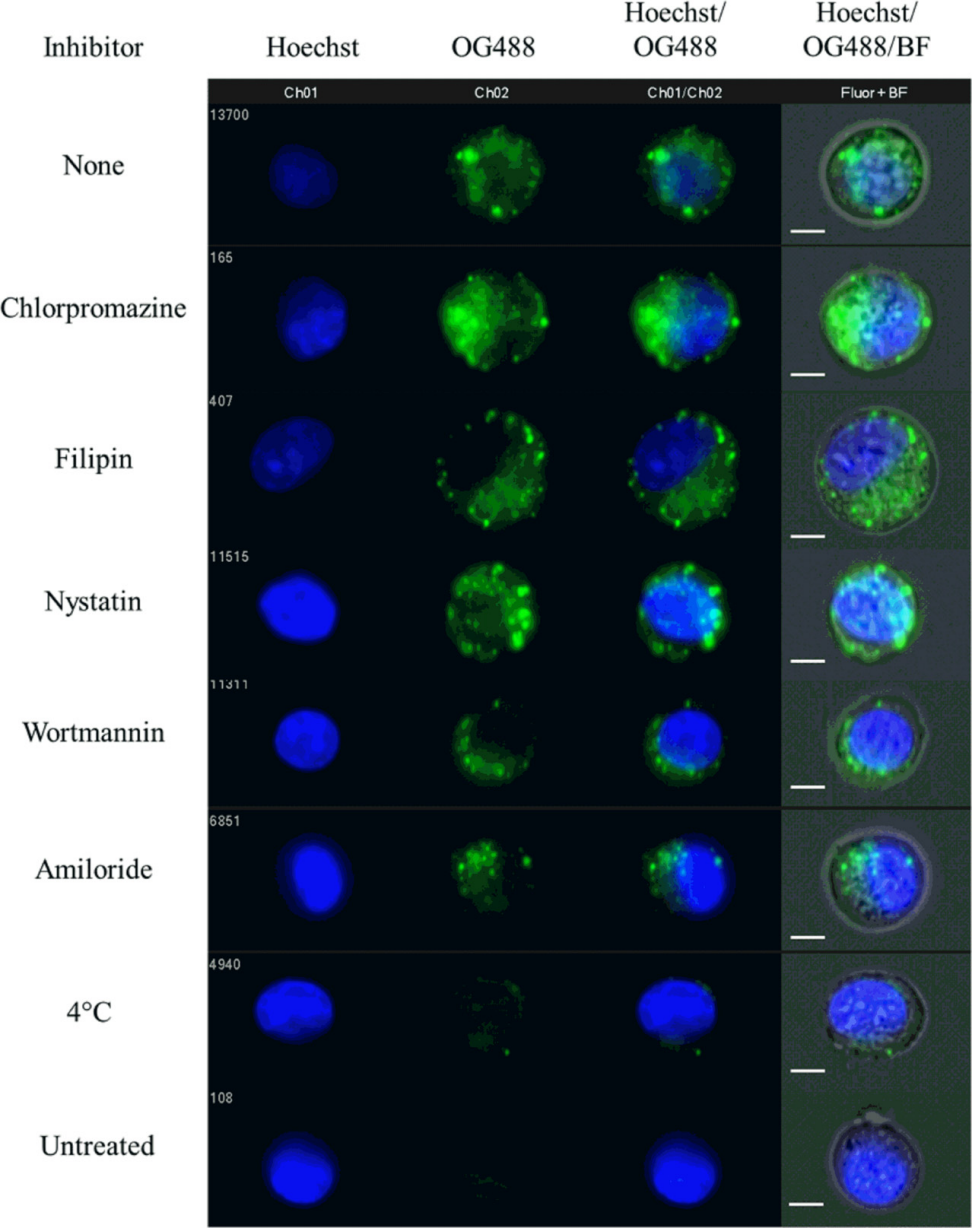
Representative fluorescent micrographs of Caco‐2 cells exposed to endocytosis inhibitors and PDETB30‐OG488. Images sampled from median intensity region of OG488 fluorescent histogram. Scale bar represents 7 μm

Figure 2 shows the intracellular fluorescence of PDETB30‐OG488 (relative to uninhibited controls) in Caco‐2 cells. Corresponding fluorescent and brightfield micrographs are seen in Figure 3. Incubation with chlorpromazine did not have a significant effect on the uptake of PDETB30‐OG488, indicating that clathrin‐mediated endocytosis is not a dominant uptake pathway for these nanogels in Caco‐2 cells. Inhibitors of caveolae‐mediated endocytosis, Filipin III and Nystatin, resulted in a 14 and 12%, respectively, reduction in intracellular fluorescence of PDETB30‐OG488. Inhibitors of macropinocytosis, wortmannin, and amiloride, resulted in the greatest decrease in intracellular fluorescence. Wortmannin caused a 39% reduction in intracellular fluorescence and amiloride caused a 31% reduction.

Inhibition of energy‐dependent processes by incubation at 4 °C caused a 63% reduction in the intracellular fluorescence. Notably, an appreciable portion of PDETB30‐OG488 uptake in Caco‐2 cells occurs through an energy‐independent process. Other reports have noted energy‐independent transport of nanoparticles, specifically with respect to cationic lipids and breast cancer cells^42^ and PLGA nanoparticles and Caco‐2 cells.^43^ This process is thought to be due to particle fusion with the cell membrane and has been reported in several types of cationic delivery vectors, including lipoplexes,^44^ dendrimers,^45^ and crosslinked poly(ethyleneimine) nanogels.^46^ These results indicate that PDETB30 is mildly membrane‐disruptive under physiological conditions and may permit the cellular influx of nanogel particles through the transient membrane perturbation and nanopore formation.

For intracellular delivery, it is important not only the uptake of the nanogels, but the number of them that are internalized. Hence, intracellular punctate staining of PDETB30‐OGG488 was analyzed in a semiquantitative fashion using Amnis IDEAS software. The intracellular spot mask was created by identifying staining patterns with spot pixel values at least 10 times greater than the surrounding cell background. A software algorithm was used to count the number of spots per cell. A demonstrative example of 0 spots, 3 spots (low spot count), and 9 spots (high spot count) is shown in Supporting Information Figure S4.

**Figure 4:**
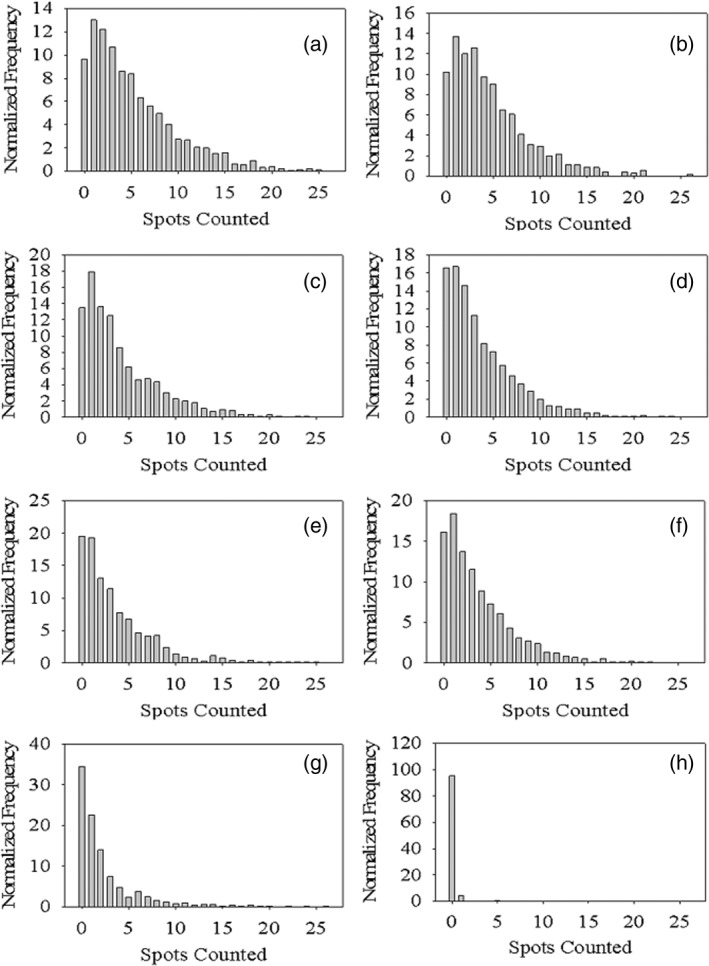
Frequency distributions of intracellular staining of PDETB30‐OG488 in Caco‐2 cells. Cellular internalization examined in the presence of no inhibitor (a), chlorpromazine (b), filipin III (c), nystatin (d), wortmannin (e), amiloride (f), or 4 °C (g). Untreated (no PDETB30‐OG488) is shown in panel (h). Caco‐2 cells were preincubated with inhibitors for 30 min, exposed to 25 μg ml^−1^ PDETB30‐OG488 for 60 min, and imaged via ImageStream cytometry after 60 min further incubation. Histograms generated from image analysis of at least 500 cells

Figure 4 shows the spot count distributions for Caco‐2 cells. This analysis reveals limited insight into the subcellular fate of PDETB30‐OG488 as a function of uptake mechanism. The number of counted spots is primarily a function of population fluorescent intensity. For example, when macropinocytosis of PDETB30‐OG488 is inhibited by amiloride in Caco‐2 cells (Figure 4, panel f) the number of counted spots decreased relative to uninhibited uptake (Figure 4, panel a).

Following the uptake analysis of our cationic nanogels that shows the internalization of these delivery vehicles, the evaluation of their membrane destabilizing capabilities was performed. This series of experiments was constructed to identify nanogels capable of selective membrane destabilization. An optimal nanogel would be relatively inert and nondisruptive under normal physiological conditions. Upon transition to endosomal conditions, this optimal nanogel would undergo a conformational transition to render it capable of potent membrane destabilization. Conversely, a nonoptimal nanogel would mediate membrane disruption under physiological conditions and/or be nondisruptive in endosomal conditions. This assessment was carried out using three different models: a hemolysis assay, LDH leakage, and GUV disruption.

**Figure 5:**
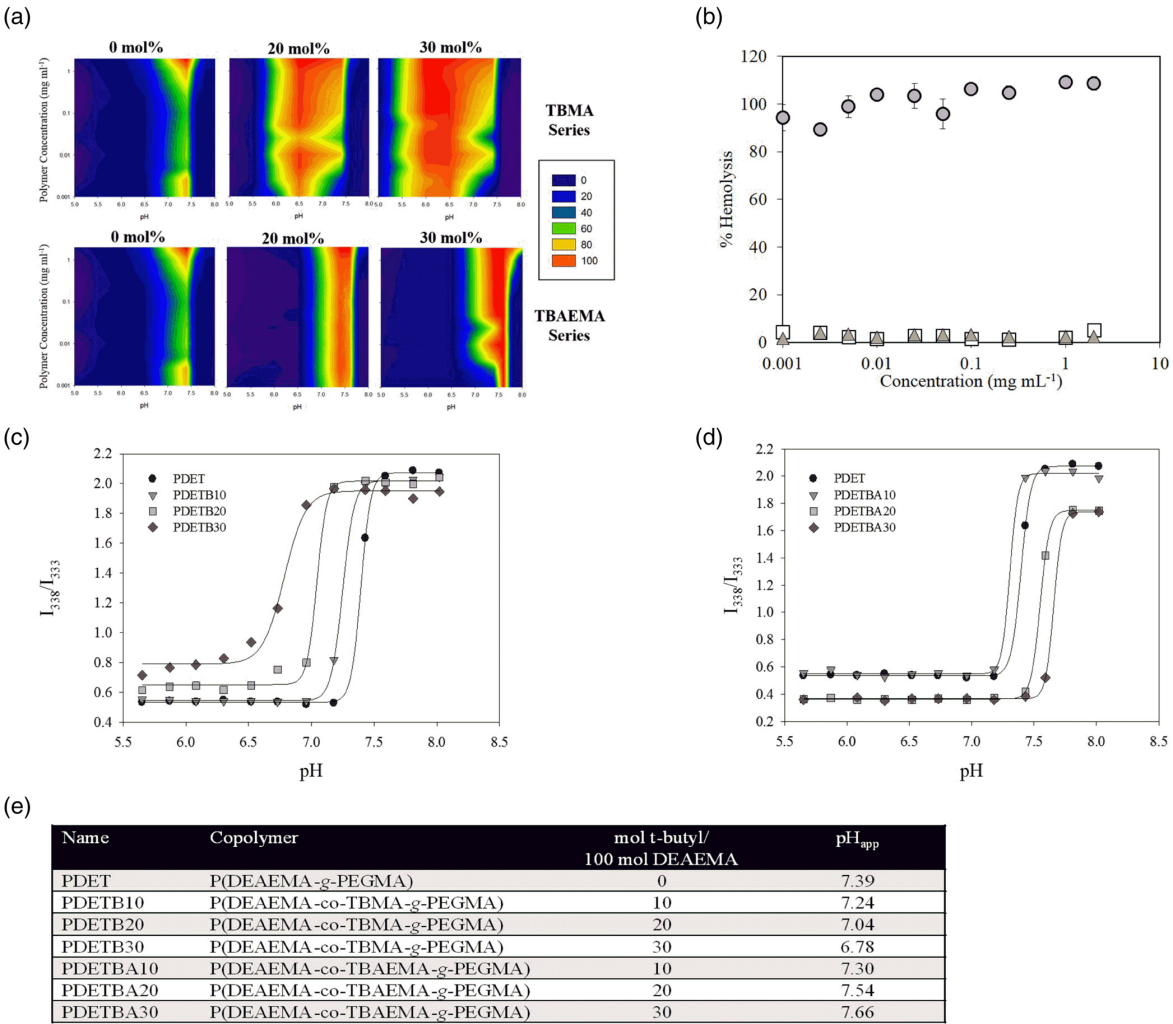
Hemolysis and pyrene fluorescence as a function of nanogel formulation and solution pH. Panel (a) shows contour plots for PDET, PDETB20, and PDETB30 (top) and PDET, PDETBA20, and PDETBA30 (bottom). Panel (b) shows the concentration‐dependent hemolytic activity of PDET (□), PDETB30 (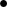), and PDETBA30 (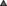) in 150 mM phosphate buffer at early endosomal pH (pH 6.0). Erythrocytes exposed to various polymer concentrations for 60 min at 37 °C. Data points represent the mean of triplicate samples ± SD. Panels (c) and (d) show the influence of TBMA incorporation on pyrene excitation (I338/I333 ratio) in P(DEAEMA‐co‐TBMA‐g‐PEGMA) nanogels (panel c), and of the inclusion of TBAEMA in the nanogel formulation (panel d). Nanogels suspended at 0.5 mg ml^−1^ and pyrene dissolved at 6 × 10–7 M in 100 mM phosphate buffers at designated pH values. Panel (e) shows a summary table of the pH transition (pH_app_) of cationic nanogel formulations

First, hemolysis experiments were used to approximate the endosomolytic ability of these nanogels. The pH‐ and concentration‐dependent hemolysis was determined according to the following equation:

$$\%\text{Hemolyis}=\frac{A_{\text{sample}}-A_{\text{blank}}}{A_{\max}-A_{\text{blank}}}$$

where *A*_sample_ represents RBCs exposed to polymer at a given pH and concentration, *A*_blank_ is the absorbance of the supernatant after RBC exposure to phosphate buffer at a given pH, and *A*_max_ represents maximum lysis following RBC exposure to DI water. The relative lysis for nanoscale hydrogels containing varying amounts of TBMA or TBAEMA is shown in contour plot form in Figure 5, panel a. These data demonstrate that polymer composition has a clear impact on membrane‐disruptive capabilities. As demonstrated previously with dynamic light scattering studies,^21^ the presence of a *t*‐butyl group alone in the copolymer is not the critical parameter for exerting control over resultant physicochemical properties. Rather, the increased network hydrophobicity of TBMA‐containing nanogels seems to govern the interactions with biological membranes.

As seen in Figure 5, inclusion of TMBA in the nanogels markedly expands both the pH and concentration range at which these networks effectively disrupt erythrocyte membranes. Hemolysis of red blood cells occurs between 7.4 and 5.5, which corresponds to the pH from physiological to late endosomal conditions. The pH transition of the synthesized formulations (as shown in Figure 5, panel e) ranges from 6.78 (PDETB30) to 7.66 (PDETBA30). The optimal formulation for intracellular delivery would have a pH transition closer to endosomal pH levels, than physiological pH. For example, PDET demonstrates efficient hemolysis at high concentrations (>0.25 mg ml^−1^) and between pH 7.0 and pH 7.6. In contrast, PDETB30 demonstrates highly efficient hemolysis in the pH range of early endosomes (pH 5.5–6.5) at concentrations as low as 1 μg ml^−1^. The enhanced hemolytic ability of PDETB30 at pH 6.0 is depicted in Figure 5, panel b, along with that of PDET and PDETBA30. Notably, PDETB30 is 10× more efficient (on a mass basis) than previously reported polycationic block copolymer systems with demonstrated efficacy in in vitro siRNA delivery^22^ and 25× more efficient than phenylalanine‐grafted pseudo‐peptides^47^ with demonstrated utility in intracellular protein delivery.^48^ These data indicate that the membrane‐disruptive properties of these nanogels can be tuned by adjusting hydrophobic monomer incorporation, an observation in accordance with several previous studies.^49^, ^50^, ^51^, ^52^, ^53^

To analyze the conformational transition of pH‐responsive nanogels, the ratio of the first to third vibronic peak (*I*_1_/*I*_3_) in the fluorescence emission spectra of pyrene was used as previously described.^21^ In the fluorescence spectra of pyrene occurs a characteristic shift depending on the polarity of the pyrene microenvironment. If dissolved in a highly polar, aqueous solvent the *I*_1_/*I*_3_ ratio in the emission spectra is approximately 1.59. This ratio decreases to 0.61 in nonpolar, aliphatic hydrocarbons such as *n‐*hexane or dodecane.^54^ Thus, a decrease in the emission *I*_1_/*I*_3_ ratio denotes the preferential partition of pyrene into hydrophobic domains.

Therefore, the pH‐responsive transition regime (from collapsed hydrophobe to swollen hydrophile) is a critical factor in determining the membrane‐disruptive ability of these nanogels. In all cases, nanogels demonstrated maximum hemolysis at or near the pH_app_ determined by pyrene fluorescence studies (Figure 5, panels c and d). If this pH_app_ is near physiological pH, this membrane‐disruptive effect was obvious in hemolysis assays (at pH 7.4). However, if the pH_app_ is decreased through increased polymer hydrophobicity (e.g., PDETB30), the nanogels are less disruptive at physiological conditions and more disruptive at endosomal conditions.

The influence of polymer composition and exposure time on membrane destabilization in live cells was further investigated using an LDH membrane integrity assay. In this assay, the percentage of LDH leakage from permeabilized or damaged cell membranes can be given by an equation analogous to:

$$\%\mathrm{LDH}\;\text{Release}=100*\frac{{\mathrm{RFU}}_{s}-{\mathrm{RFU}}_{\mathrm{PBS}}}{{\mathrm{RFU}}_{\max}-{\mathrm{RFU}}_{\mathrm{PBS}}}$$

where RFU_S_ is the fluorescent reading from the sample, RFU_PBS_ is the fluorescent reading from cells exposed only to PBS (0% lysis) and RFU_max_ (100% lysis) is the maximum fluorescent reading from the plate. In typical applications, RFU_max_ is given by a commercial lysis buffer. In practice, however, the fluorescent reading generated by the greatest polymer concentration (2 mg ml^−1^) generated fluorescent values that exceeded that of the kit lysis buffer and 1% wt/vol solutions of Triton‐X100. Thus, LDH release is occasionally reported as >100% at polymer concentrations 1–2 mg ml^−1^.

**Figure 6:**
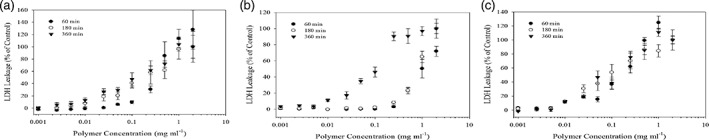
Representative time‐dependent LDH leakage from Caco‐2 cells following 60 min (●), 180 min (○), or 360 min (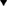) exposure to PDET (a), PDETB30 (b), and PDETBA30 (c). Data points represent the sample mean ± *SEM* (*n* = 4). LDH leakage calculated relative to untreated cells and surfactant‐lysed cells

LDH leakage as a function of nanogel concentration and exposure time is shown in Figure 6 for PDET (panel a), PDETB30 (panel b), and PDETBA30 (panel c). For PDET (Figure 6, panel a), the LDH leakage increases with longer exposure time (60–180 min) and remains relatively constant from 180 to 360 min. For PDETB30 (Figure 6, panel b), the LDH leakage is negligible at concentrations up to 250 μg ml^−1^ for 60 and 180 min exposure. However, the leakage increases considerably following 360 min exposure. LDH release following exposure to PDETBA30 (Figure 6, panel c) follows no clear time dependence and the release values are similar across all time points. These data underscore the need for careful consideration of incubation time in future cytotoxicity and drug delivery experiments to minimize the nonselective disruption of cellular membranes.

**Figure 7:**
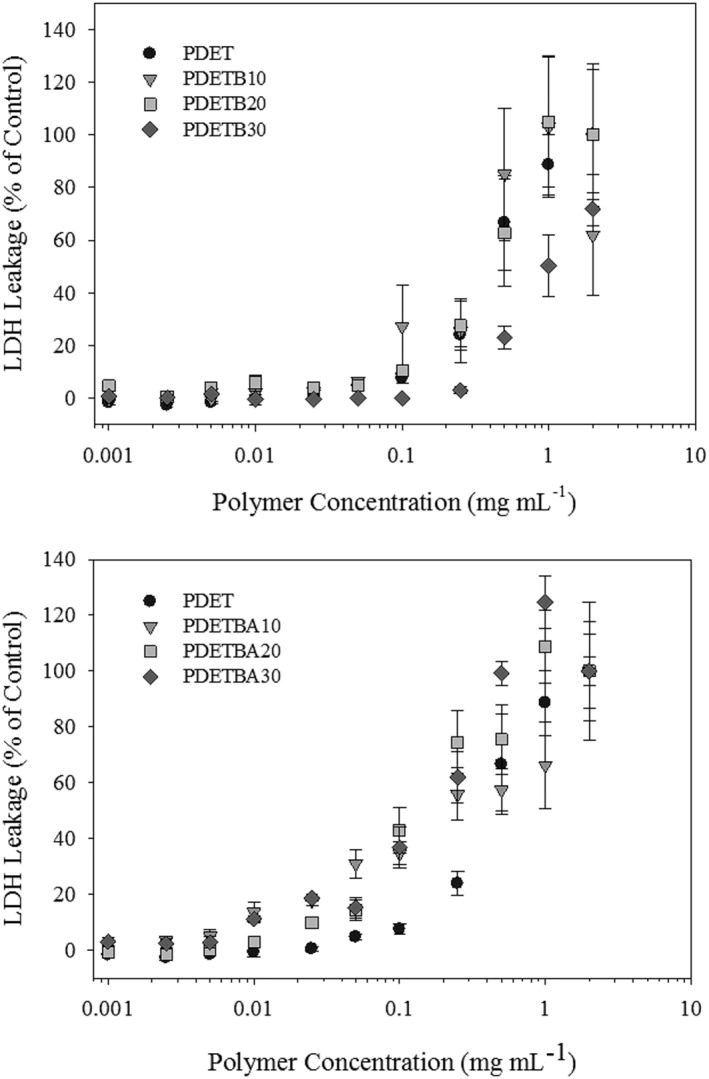
Polymer‐mediated LDH leakage from Caco‐2 cells following exposure to PDET (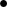), PDETB10 (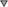), PDETB20 (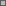), or PDETB30 (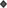) for 60 min (a) or PDET (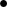), PDETBA10 (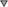), PDETBA20 (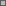), or PDETBA30 (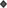) for 60 min (b)

The influence of nanogel composition on LDH leakage, shown in Figure 7 for TBMA‐containing polymers (panel a) and for TBAEMA‐containing polymers (panel b), show that PDETB30 is less damaging to Caco‐2 cell membranes than PDET, PDETB10, and PDETB20. The general trend for inducing LDH membrane leakage is PDET ~ PDETB10 ~ PDETB20 > PDETB30, showing that the higher amount of TBMA incorporation reduces the damage to Caco‐2 cells. For the TBAEMA‐containing polymers (Figure 7, panel b), the general trend is as follows: PDETBA30 > PDETBA20 ~ PDETBA10 > PDET. Notably, these trends are in excellent agreement with the trends in hydrophobic–hydrophilic phase transition as demonstrated using pyrene fluorescence measurements shown in Figure 5.

Visualizing a model lipid bilayer during the destabilization can provide some insight into the mechanism of membrane disruption. Prevailing theories for membrane disruptive mechanisms by cationic polymers include reorientation of lipid head groups through ammonium–phosphate interactions,^55^ transient nanopore formation^56^, ^57^ following electrostatic attraction between polycation and cell membrane, or even catastrophic membrane disruption.^58^ Naturally, size, surface charge, and ligand functionalization play important roles in modulating membrane interaction.^59^ Many of these studies rely on biophysical measurements of controlled model systems such as supported lipid bilayers. Conversely, mammalian cell membranes typically contain dynamic combinations of surface‐ and transmembrane proteins, sugar coatings, diverse lipid combinations, and cholesterol, which increase the complexity of the membrane interactions.

**Figure 8:**
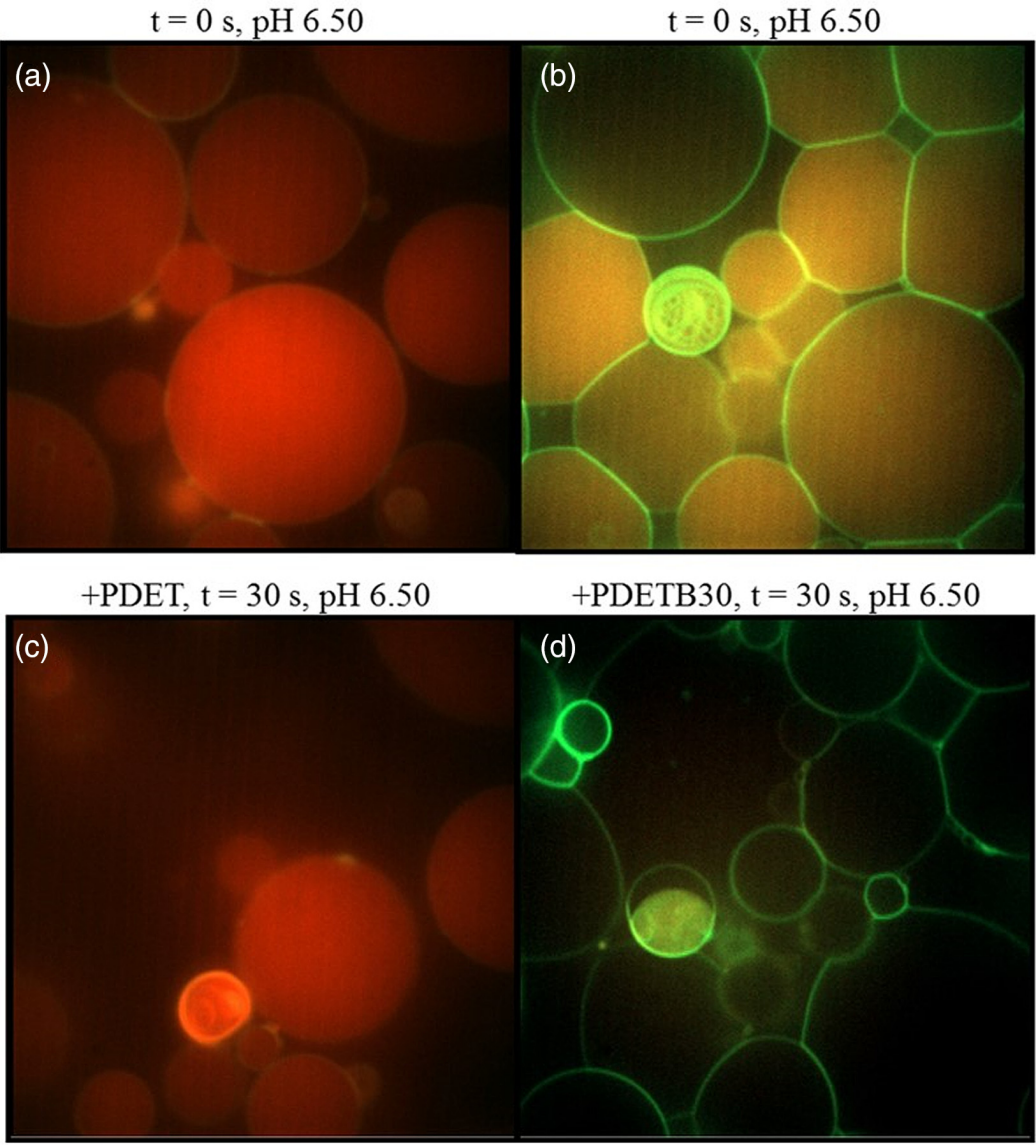
Destabilization of GUV membranes. Intravesical red fluorescence indicates sucrose‐Texas Red. Green fluorescence indicates membrane lipid DHPE‐Bodipy FL. GUVs were suspended in 100 mM phosphate buffer at pH 6.5. PDET (a) or PDETB30 (b) in isosmotic phosphate buffer was added at achieve a final concentration of 50 μg ml^−1^. GUVs after 30 s incubation (c and d). Images captured using Zeiss spinning disc confocal microscope at 100×

The micrographs in Figure 8 suggest that transient nanopore formation is the predominant mechanism through which PDETB30 exerts a membrane‐destabilizing effect. For these initial studies, pH 6.50 was selected to approximate the pH of an early endosomal environment. Based on the hemolysis studies presented in Figure 5, PDET should be nondisruptive and PDETB30 should be highly‐disruptive at these conditions. Following an injection to bring the PDET to 50 μg ml^−1^ in the buffered GUV solution, no discernible change was detected in membrane integrity. The sucrose‐Texas Red remains entrapped in the GUV for several minutes after injection, confirming the persistence of membrane integrity as shown in panels a and c.

In contrast, the micrographs in Figure 8, panels b and d, reveal substantial PDETB30‐mediated destabilization of lipid membranes. Exposure to 50 ug ml^−1^ PDETB30 in a pH 6.50 buffer solution resulted in a rapid and complete efflux of sucrose‐Texas Red from the vesicle interior. These data concur with the hemolysis data for PDETB30 at this concentration and pH, which indicate complete (~100%) disruption of erythrocytes. Further efforts in this area may help determine if the mechanism of membrane destabilization exhibits a dependence on polymer concentration.

## 4 CONCLUSIONS

Physicochemical properties of nanoscale hydrogel networks, including critical phase transition pH, membrane disruption, and cytocompatibility can be modulated by tuning polymer composition. The mechanisms of cellular internalization of fluorescent nanogels were studied using imaging flow cytometry in Caco‐2 cells, which showed that despite the lack of any targeting moieties, these nanogels are readily taken up. After 60 min exposure, the intracellular PDETB30‐OG488 fluorescence increased over 25× in treated cells. Additionally, this analysis also showed that macropinocytosis is the dominant mechanism of nanogel internalization in a model cell line. Membrane vesicles arising from clathrin‐mediated endocytosis and macropinocytosis both undergo acidification. As PDETB30 requires a slightly acidic pH to exert its membrane‐destabilizing effects, these internalization pathways are desirable for uptake and subsequent endosomal escape of PDETB30 and encapsulated therapeutics. Additionally, the breadth of the pH range for maximum membrane disruption is related to the pH range for hydrophobic‐hydrophilic transition. In particular, PDETB30 is membrane‐disruptive over a broader pH range than other nanogels that undergo a more rapid hydrophobic‐hydrophilic phase transition (e.g., PDET and PDETBA30). For these reasons, we have shown that TBMA‐containing nanogels exhibit favorable pH‐responsive phase transition behavior for intracellular delivery and offer an excellent combination of cytocompatibility, hemolytic ability, and membrane‐disruptive properties. These characteristics are crucial to their promise as intracellular drug delivery vehicles.

## Supporting information


**Supplementary Figure S1.** Representative transmission electron micrographs of cationic pH‐responsive nanogels PDET (A) and PDETB30 (B). Particles stained with uranyl acetate and images collected at 43,000. Scale bar represents 200 nm.
**Supplementary Figure S2.** Cytotoxicity of inhibitors on Caco‐2 cells following 90 min exposure. Cellular proliferation relative to untreated control was determined via MTS assay. Data represent the mean of quadruplicate samples ±s.e.m. Dashed vertical line designates the concentration used in inhibition studies.
**Supplementary Figure S3.** Histogram of the internalization coefficient of fluorescent nanogels. Fluorescent intensity of PDETB30‐OG488 in uptake inhibition studies was calculated from cells with internalization coefficient > 0.
**Supplementary Figure S4.** Spot counting of intracellular nanogels. Left images show fluorescent intensity (white) of fluorescently‐labeled PDESSB30‐OG488. Three representative images of low (1 spot) count (A), intermediate (5 spots) count (B), and high (9 spots) count (C). Spot masks are shown in turquoise overlaid against high‐intensity areas in the fluorescent image.
**Supplementary Table S1.** Polymeric cationic nanoparticle characterization. Polymer composition, size, and particle charge of the synthesized nanogels. Nomenclature of the synthesized nanogels is based on the theoretical content of hydrophobic monomer in the resulting polymer. Diameters of dry nanogels were calculated from TEM micrographs. Values reported represent the mean ± s.d. (n > 150). Effective surface zeta‐potential of polymer formulations synthesized with different amounts of TBMA or TBAEMA. Values reported represent the mean of 10 measurements. Adapted from reference 21.
