# Supplementary material for: Uptake and function of membrane‐destabilizing cationic nanogels for intracellular drug delivery
Source: Bioeng Transl Med. 2018 Nov 22;4(1):17–29. doi: 10.1002/btm2.10120 (PMC6336667; doi:10.1002/btm2.10120)
Supplement: Supplementary file 1 — Supplementary Figure S1. Representative transmission electron micrographs of cationic pH‐responsive nanogels PDET (A) and PDETB30 (B). Particles stained with uranyl acetate and images collected at 43,000. Scale bar represents 200 nm. Supplementary Figure S2. Cytotoxicity of inhibitors on Caco‐2 cells following 90 min exposure. Cellular proliferation relative to untreated control was determined via MTS assay. Data represent the mean of quadruplicate samples ±s.e.m. Dashed vertical line designates the concentration used in inhibition studies. Supplementary Figure S3. Histogram of the internalization coefficient of fluorescent nanogels. Fluorescent intensity of PDETB30‐OG488 in uptake inhibition studies was calculated from cells with internalization coefficient > 0. Supplementary Figure S4. Spot counting of intracellular nanogels. Left images show fluorescent intensity (white) of fluorescently‐labeled PDESSB30‐OG488. Three representative images of low (1 spot) count (A), intermediate (5 spots) count (B), and high (9 spots) count (C). Spot masks are shown in turquoise overlaid against high‐intensity areas in the fluorescent image. Supplementary Table S1. Polymeric cationic nanoparticle characterization. Polymer composition, size, and particle charge of the synthesized nanogels. Nomenclature of the synthesized nanogels is based on the theoretical content of hydrophobic monomer in the resulting polymer. Diameters of dry nanogels were calculated from TEM micrographs. Values reported represent the mean ± s.d. (n > 150). Effective surface zeta‐potential of polymer formulations synthesized with different amounts of TBMA or TBAEMA. Values reported represent the mean of 10 measurements. Adapted from reference 21. [file BTM2-4-17-s001.docx]

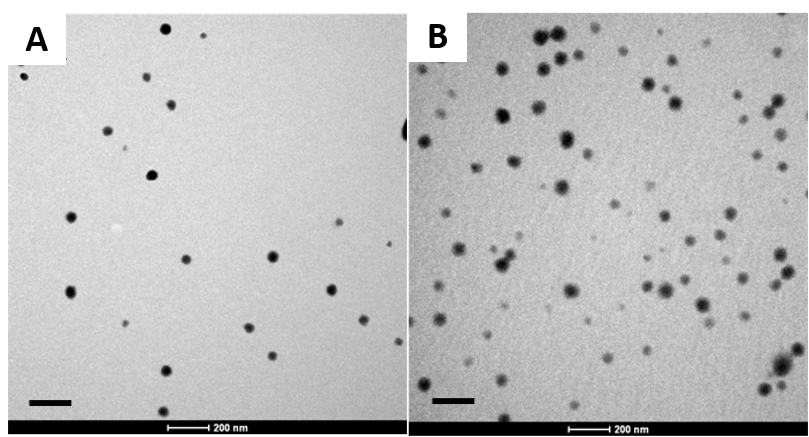


**Supplementary Figure 1. Representative transmission electron micrographs of cationic pH-responsive nanogels PDET (A) and PDETB30 (B).** Particles stained with uranyl acetate and images collected at 43,000. Scale bar represents 200 nm.





**Supplementary Figure 2. Cytotoxicity of inhibitors on Caco-2 cells following 90 min exposure.**  Cellular proliferation relative to untreated control was determined via MTS assay. Data represent the mean of quadruplicate samples ±s.e.m. Dashed vertical line designates the concentration used in inhibition studies.





**Supplementary Figure 3. Histogram of the internalization coefficient of fluorescent nanogels.**  Fluorescent intensity of PDETB30-OG488 in uptake inhibition studies was calculated from cells with internalization coefficient > 0.





**Supplementary Figure 4. Spot counting of intracellular nanogels.**  Left images show fluorescent intensity (white) of fluorescently-labeled PDESSB30-OG488. Three representative images of low (1 spot) count (A), intermediate (5 spots) count (B), and high (9 spots) count (C). Spot masks are shown in turquoise overlaid against high-intensity areas in the fluorescent image.

| Name | Polymer | mol *t*-butyl/ 100 mol DEAEMA | Calculated Diameter (dry nanogels) | Zeta Potential Average (mV) |
| --- | --- | --- | --- | --- |
| PDET | P(DEAEMA-g-PEGMA) | 0 | 47 ± 13 | 13 |
| PDETB10 | P(DEAEMA-co-TBMA-g-PEGMA) | 10 | 60 ± 26 | 16 |
| PDETB20 | P(DEAEMA-co-TBMA-g-PEGMA) | 20 | 50 ± 22 | 8 |
| PDETB30 | P(DEAEMA-co-TBMA-g-PEGMA) | 30 | 52 ± 17 | 5 |
| PDETBA10 | P(DEAEMA-co-TBAEMA-g-PEGMA) | 10 | 50 ± 18 | 11 |
| PDETBA20 | P(DEAEMA-co-TBAEMA-g-PEGMA) | 20 | 63 ± 21 | 13 |
| PDETBA30 | P(DEAEMA-co-TBAEMA-g-PEGMA) | 30 | 66 ± 25 | 11 |

**Supplementary Table 1. Polymeric cationic nanoparticle characterization.**  Polymer composition, size, and particle charge of the synthesized nanogels. Nomenclature of the synthesized nanogels is based on the theoretical content of hydrophobic monomer in the resulting polymer. Diameters of dry nanogels were calculated from TEM micrographs. Values reported represent the mean ± s.d. (n > 150). Effective surface zeta-potential of polymer formulations synthesized with different amounts of TBMA or TBAEMA. Values reported represent the mean of 10 measurements. Adapted from reference 21.
